# Insights into an Original Pocket-Ligand Pair Classification: A Promising Tool for Ligand Profile Prediction

**DOI:** 10.1371/journal.pone.0063730

**Published:** 2013-06-20

**Authors:** Stéphanie Pérot, Leslie Regad, Christelle Reynès, Olivier Spérandio, Maria A. Miteva, Bruno O. Villoutreix, Anne-Claude Camproux

**Affiliations:** 1 INSERM, UMRS 973, MT*i*, Paris, France; 2 Univ Paris Diderot, Sorbonne Paris Cité, UMRS 973, MT*i*, Paris, France; University of Alberta, Canada

## Abstract

Pockets are today at the cornerstones of modern drug discovery projects and at the crossroad of several research fields, from structural biology to mathematical modeling. Being able to predict if a small molecule could bind to one or more protein targets or if a protein could bind to some given ligands is very useful for drug discovery endeavors, anticipation of binding to off- and anti-targets. To date, several studies explore such questions from chemogenomic approach to reverse docking methods. Most of these studies have been performed either from the viewpoint of ligands or targets. However it seems valuable to use information from both ligands and target binding pockets. Hence, we present a multivariate approach relating ligand properties with protein pocket properties from the analysis of known ligand-protein interactions. We explored and optimized the pocket-ligand pair space by combining pocket and ligand descriptors using Principal Component Analysis and developed a classification engine on this paired space, revealing five main clusters of pocket-ligand pairs sharing specific and similar structural or physico-chemical properties. These pocket-ligand pair clusters highlight correspondences between pocket and ligand topological and physico-chemical properties and capture relevant information with respect to protein-ligand interactions. Based on these pocket-ligand correspondences, a protocol of prediction of clusters sharing similarity in terms of recognition characteristics is developed for a given pocket-ligand complex and gives high performances. It is then extended to cluster prediction for a given pocket in order to acquire knowledge about its expected ligand profile or to cluster prediction for a given ligand in order to acquire knowledge about its expected pocket profile. This prediction approach shows promising results and could contribute to predict some ligand properties critical for binding to a given pocket, and conversely, some key pocket properties for ligand binding.

## Introduction

Being able to predict if small organic compounds could bind to one or more protein targets or if a protein could bind to some given ligands would be very valuable to drug discovery and chemical biology projects alike. Among the *in silico* approaches that have been developed to assist such investigation, one can distinguish ligand-based and structure-based methods [1]. These prediction methods are in part based on two principles that are only partially true: similar chemical structures tend to present similar biological activity and similar receptors are supposed to bind similar ligands [2], [3].

Several concepts and algorithms are being used to explore such questions and include among other chemogenomic approaches that attempt to fully match target and ligand space, through methods in which targets are classified, not according to sequence or fold, but according to the similarity of ligands or *via* reverse docking where ligands are positioned in a library of pockets [4]–[8]. Pharmacophore models based on the 3D structures of protein-ligand complexes can also be used [9] as well as hybrid 2D/3D target prediction methods such as ReverseScreen3D [10]. Comparison of binding pockets can be carried out with the aim of exploring the relationship with the corresponding ligands requiring or not the 3D structures of the targets [11]–[14]. To date, most of the research has been performed separately either from the viewpoint of ligands or targets. However, whenever possible, it would be valuable to use information from both ligands and target binding cavities. Proteochemometric modeling has been developed along this line and can be defined as a tool to extrapolate from the activities of known ligands for known target to novel targets and conversely to virtually screen for selective compounds that are solely active on a single member of a subfamily of targets [15], [16], [17]. By contrast to traditional QSAR approaches usually based only on the ligand space, proteochemometric modeling is based on the similarity of a group of ligands and a group of targets, such as to investigate the so-called ligand-target interaction space [18]. The advantage of merging ligand and target information is illustrated in the study of Weill and Rognan [19] where they propose a model with a homogeneous cavity description applied to a unique family of targets (GPCR). They conclude unambiguously that protein-ligand fingerprints outperform the corresponding ligand fingerprint in predicting either putative ligands for a known target or putative targets for a known ligand. Similarly, Yamanishi *et al.* [14], develop a novel method to extract sets of drug chemical substructures and protein domains that govern drug-target interactions on a genome-wide scale. Their method is based only on protein domain information, *i.e.* without considering the 3D structure of the target, and could help to eliminate molecules with too many potential off-target interactions or with specific off-targets expected to lead to severe side effects. Meslamani *et al.* [20] have recently suggested that taking into account true 3D cavity descriptors rather than simple sequence-based target slightly enhances the accuracy of their models to discriminate true target-ligand complexes from false pairs. However, much work is still needed to perform polypharmacology profiling or to predict off-target interactions [5]. There are several proteochemometric modeling difficulties such as introduction of a large number of parameters to model both molecule and binding site spaces, which may reduce model performance, or ensuring that both compound and protein descriptors are compatible and interpretable [21]–[22].

Taken together, the concepts and studies mentioned above demonstrated that considering ligand and pocket descriptors together is of major importance and prompt us to propose a new description of the protein-ligand interface by merging, optimizing and combining descriptors of the protein-ligand pair space. We want to investigate the development of a method that relates ligand characteristics with protein pocket characteristics at first within the framework of known ligand-protein interactions. In this paper, we propose an original method to model and explore what we call the pocket-ligand pair space. For this purpose, we used a dataset of co-crystallized pockets and ligands and computed descriptors on both pockets and ligands. We then developed a classification engine proceeding on this pocket-ligand pair space obtained through a Principal Component Analysis (PCA), which helps the exploration and optimization of the pocket-ligand pair space. The structural and physico-chemical similarities between the pocket-ligand pairs are then visualized in a clustering tree that reveals five main clusters of pocket-ligand pairs sharing specific and similar topological and physico-chemical characteristics. These pocket-ligand pair clusters are meaningful and capture similarity in terms of recognition characteristics that can be relevant information with respect to protein-ligand interaction properties. Our classification can provide information about the profile of a putative ligand for a given pocket and conversely. Taken these five main clusters as reference classes, we then propose a cluster prediction method. The method enables first to assign a new pocket-ligand pair to one cluster and then to propose potential partner characteristic profile by assigning a ligand or a pocket to one of the clusters.

## Results and Discussion

### Determination of Pocket-ligand Pair Space Characteristics

A multivariate analysis on pocket-ligand pair space was used to take into account the interaction between a target and a ligand. This was performed by combining both the physico-chemical and geometric properties of pocket and ligand descriptors extracted from a large *training* set of 483 complexes from the PDBbind and Astex datasets.

The first step consisted to remove most redundant descriptors amongst the 24 considered pocket descriptors and amongst the 20 considered ligand descriptors but keeping the most informative pocket-ligand properties involved in the interaction using correlation parameter (see Materials and Methods). Typically, when several pocket descriptors are strongly correlated, the one being also relevant on ligands is chosen to favor correspondences between pocket and ligand spaces. Similarly, if several ligand descriptors are strongly correlated, the one that can be computed on pockets is kept. For instance, sphericity descriptor exhibits a strong negative correlation with volume (−0.77, see Figure S1.A), thus only the volume descriptor, easily calculated and interpretable for both pockets and ligands, is considered for the analysis. Conversely roughness descriptor exhibits weak correlation with volume one (−0.28, see Figure S1.B) and could give complementary information to volume data and is therefore kept.

**Figure 1:**
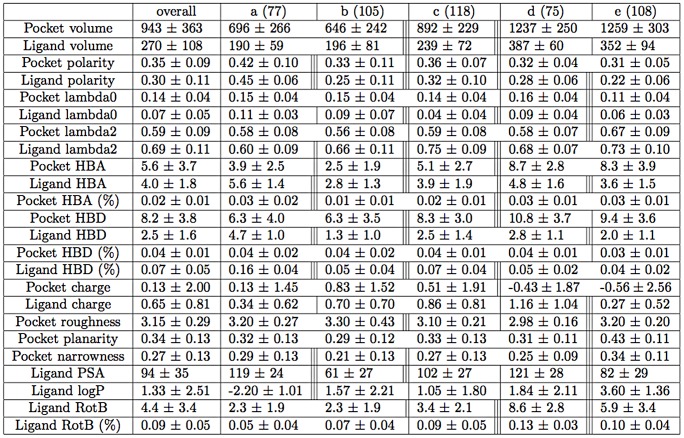
Averages and standard deviations of main pocket and ligand descriptors for the five clusters a, b, c, d and e. For the five clusters, if the corresponding analysis of variance and the Tukey’s HSD test are significant, a vertical line between two clusters is drawn. No vertical line means that the test is not significant *i.e.* the p-value is more than 0.05. One, two or three vertical lines mean that the test is significant and that: 0.01≤p-value≤0.05 for one vertical line, 0.001≤p-value≤0.01 for two vertical line or p-value≤0.001 for three vertical lines.

At the end of this descriptor selection protocol, there are 24 descriptors left with 8 computed on both pockets and ligands: volume, polarity, moments of inertia one and three (lambda0, lambda2), charge, hydrogen bond donor counts and proportions (respectively, HBD and hbd%), hydrogen bond acceptor counts (HBA), 4 descriptors to describe more precisely the pocket topology: roughness, planarity, narrowness, hydrogen bond acceptor proportions (hba%) and 4 descriptors to describe the ligand: rotatable bond counts and proportions (RBond, rbond%) and which 2 describing the ligand polarity (polar surface area (PSA), LogP. The average value and standard deviation of these 24 descriptors are given in Figure 1 (first column). These average values are in agreement with several typical features already observed in previous studies. For instance, our average pocket volume (943 Å^3^) is close to the one observed by Nayal *et al.* [23] (930 Å^3^), despite the fact that binding site description is depending both on datasets and methods used to compute pocket volume. Another important feature taken into account is polarity since polar interactions play a crucial role in the binding. We observe here similar polarity values on average for pockets and ligands (respectively, 0.35±0.1 for pockets and 0.30±0.1 for ligands) in agreement with the pocket polarity ratio estimated in the study of Eyrisch *et al.* [24], which lies between 0.25 and 0.45. The observed average pocket roughness in our study, 3.15±0.29, is in agreement with Pettit *et al.* [25] who reported an average value of 3.28±0.10.

Concerning pocket-ligand descriptors, there is a significant positive correlation (0.69) between their respective volume: the pocket volume is more than three times larger than the ligand volume (see Figure S1.C) in agreement with Liang *et al.* [26] and with the gap between ligands and pockets explained by the existence of a “buffer zone” or region of free space between ligands and pockets as also mentioned by Kahraman *et al.* [27]. Pocket and ligand volume correlation is fuzzier for larger pockets, which bind both large and medium size ligands. Pocket and ligand polarities are positively correlated (0.53): small polar ligands are associated to small polar pockets but more polar ligands are possibly associated to more or less polar pockets (see Figure S1.D). Some further analysis about the role of water molecules in binding pocket would be required when discrepancy is observed but is beyond the scope of the present study. Finally, a multivariate analysis is performed to propose interpretable analysis combining all these pocket and ligand descriptors in a more complex and informative way.

### Description of the Pocket-ligand Pair Space

**Figure 2:**
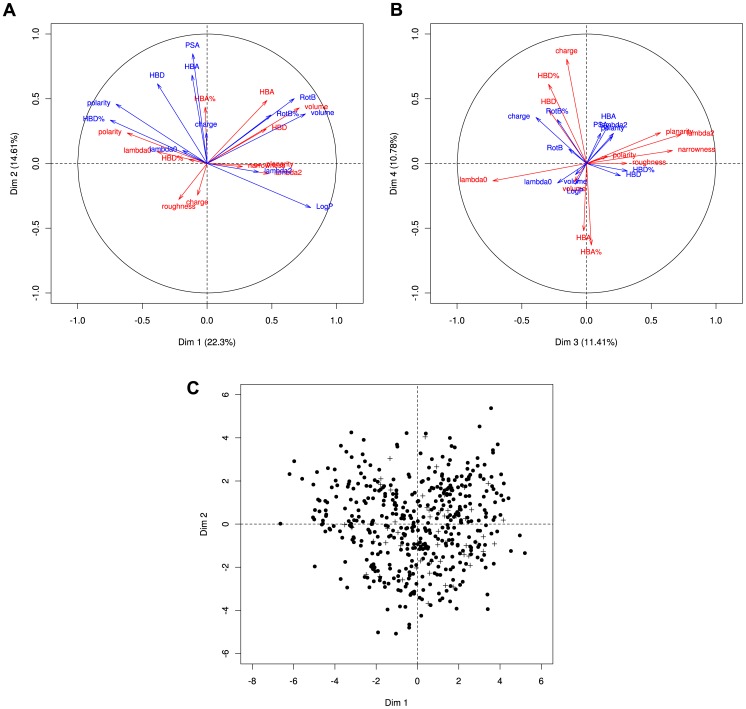
Principal Component Analysis (PCA) of pocket-ligand pair space. Ligand and pocket descriptors are indicated by respectively red and blue arrows in (A) and (B). Closer are the descriptors to the correlation circle, more they contribute to explain the variability captured by the corresponding principal components. (A) corresponds to the first principal component (PC1) that captures 22.3% of the variability *versus* second principal component PC2 (14.6%). (B) corresponds to the plot of the third principal component PC3 (11.4%) *versus* the fourth one PC4 (10.7%). These two planes capture a total of 59% of the variability of the data. (C) PCA of pocket-ligand pairs from the *training* set are depicted in black points with Astex dataset indicated by “+”. This illustrates Astex dataset is well sampled on the first plane with no specific characteristic in terms of the 24 considered descriptors.

PCA performed on the 24 pocket and ligand descriptors enables an in-depth study of the properties shared by similar pairs by providing a suitable representation of our pocket-ligand pairs. The four main components are illustrated in Figure 2.A, B. The first two components (PC1 and PC2) capture more than 36% of the data variability and the first four ones around 60%, and as such capture a large part of the variability of the data. As indicated by the projection of the variables close to the PCA correlation circle, different selected pocket and ligand descriptors contribute to the variability of the data and are relevant for the study. Figure 2.A summarizes the correlations between descriptors and the first principal plane (PC1+PC2). Pocket and ligand polarities are strongly negatively correlated related to PC1 whereas the pocket and ligand volumes are strongly positively correlated related to PC1.Pocket and ligand pairs having high values for PC1 tend to be larger and slightly polar while they tend to be smaller and more polar for small values of PC1. For instance, pocket-ligand pairs with a PC1 value higher than 3 correspond to pockets with an average volume and polarity of 1451 Å^3^ (±337) and 0.30 (±0.06), respectively. Whereas pocket-ligand pairs with a PC1 value smaller than −4 correspond to pockets with an average volume and polarity of 505 Å^3^ (±193) and 0.5 (±0.09), respectively. Interestingly, other descriptors also contribute to these main components. Large volume and weak polarity values of pockets and ligands are associated to high LogP, rbond% and weak hbd% ligand values. PC2 is explained by other ligand descriptors such as PSA, HBD and HBA (proportions and counts). From top to bottom of the PC2, ligand PSA, HBD and HBA values tend to decrease while pocket roughness values tend to increase. This suggests that pockets with high roughness values tend to bind ligands with weak PSA, HBA and HBD values, and therefore a lower polarity. The third principal component (PC3), which captures 11.4% of the variability, is mainly explained by shape pocket descriptors such as moments of inertia, planarity and narrowness whereas the fourth principal component (PC4), which captures 10.7% of the variability, is mainly explained by pocket HBA and HBD values, as illustrated in Figure 2.B.

This PCA analysis defining the pocket-ligand pair space is in agreement with results obtained on the pocket space [28] or on the ligand space [29], [30]. Concerning the pocket space, we observe that small pockets tend to be more polar and to bind small and polar ligands, as previously observed [25], [27], [31]–[33]. These descriptors are in agreement with a PCA model developed in [28], which indicates that size and shape descriptors are the most important properties followed by polarity and charge. Moreover, Figure 2.C shows that drug-like molecules (corresponding to Astex dataset is well sampled on the PCA first plane. These results are in agreement with Oprea *et al.* [29] who characterized the chemical space of drug-like ligands using PCA model and showed that size and shape are the most significant properties followed by lipophilicity then the flexibility and polarity of ligands.

Hence, one can observe that neither pocket descriptors nor ligand descriptors gather the whole information since the components are explained by both pocket and ligand descriptors combined in a complex way, validating the fact that it is more informative to take into account the pocket space and the ligand space together than independently whenever possible. Classical descriptors such as volume, shape and polarity are complementary for both pockets and ligands. Moreover, our analysis also highlights some specific properties of ligands, such as PSA, HBA, HBD, RBond, polarity corresponding to specific geometrical properties of pockets such as roughness, planarity and narrowness. All subsequent analyses will be performed on the *optimal pocket-ligand space* defined by the principal components accounting for 95% of the global variability, capturing on both pocket and ligand descriptors that is to say the first fourteen principal components.

### Clustering of Pocket-ligand Pairs

To map and group together pocket-ligand pairs with similar properties, we built a hierarchical clustering tree (see Material and Methods) on the *optimal pocket-ligand space* obtained on our set of 483 complexes.

**Figure 3:**
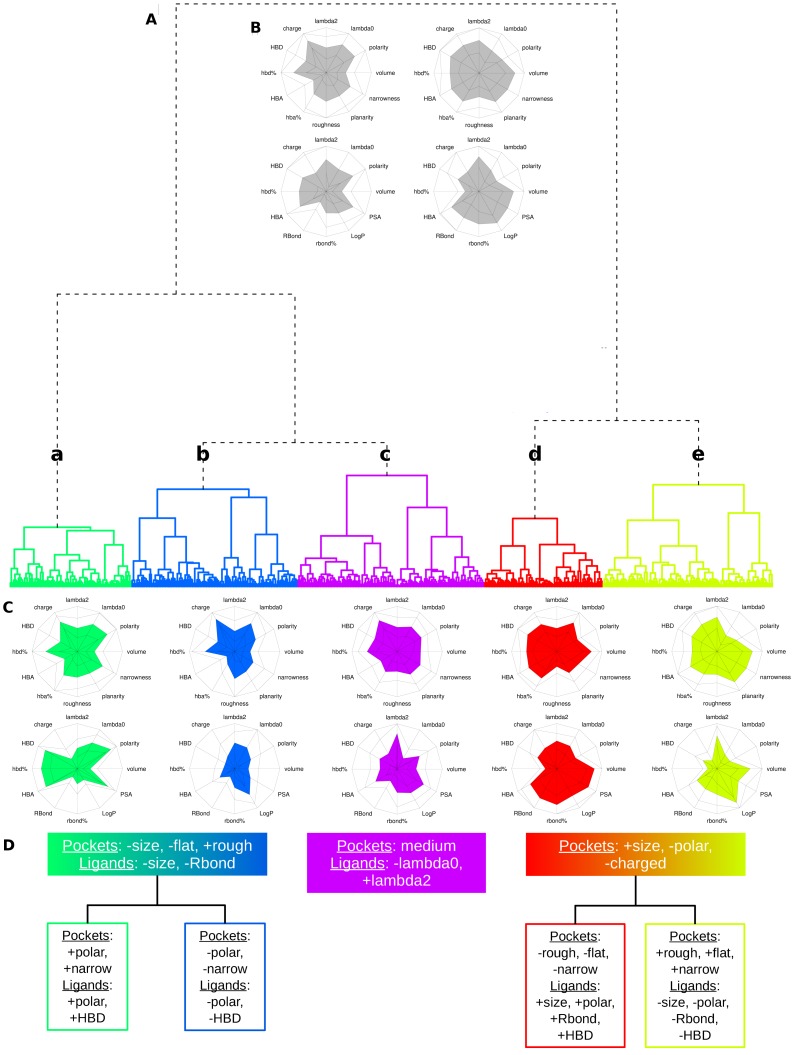
Characterization of pocket-ligand pair clusters. One color is dedicated to each cluster for all the pictures. (A) The hierarchical classification tree of pocket-ligand pairs on the *optimal pocket-ligand space* (first 14 principal components of PCA model) presented five clusters: **a**, **b**, **c**, **d** and **e**. (B) The tree is first divided into two clusters whose main pocket (top stars) and ligand (bottom stars) properties are shown by grey star-plots. The solid surface of a star-plot is limited by the average value of the considered descriptors and the black lines correspond to the standard deviations of the considered descriptors. (C) The main pocket (top stars) and ligand (bottom stars) properties of each cluster are shown by colored star-plots. Pocket descriptors correspond to the first five star-plots and ligand descriptors correspond to the last five star-plots. (D) The main pocket-ligand pair cluster properties are summarized.

Concerning the structure of the tree (Figure 3.A), pocket-ligand pairs were first split into two clusters described by average values of 12 pocket and 12 ligand descriptors on two grey star-plots, see Figure 3.B. These grey star-plots illustrate the main classical correspondences between pocket and ligand descriptors: small pockets are also polar and bind small and polar ligands, in agreement with previous observations. We note that the smaller pockets grouping in one pocket-ligand pair cluster tends to be rougher in agreement with Pettit and Bowie [25] and more polar as suggested by Eyrisch *et al.* [24] than larger pockets of other clusters.

Then, pocket-ligand pairs were split into five clusters named **a**, **b**, **c**, **d** and **e** (Figure 3.A) grouping similar pocket-ligand pairs in terms of the 24 selected descriptors, with reasonable occurrence (more than 75 pairs in each cluster). Figure 1 presents a detailed description of these five clusters **a**, **b**, **c**, **d** and **e** in terms of average and standard deviation of pocket and ligand descriptors. It also presents the results of the analysis of variance and the Tukey’s HSD *post-hoc* tests indicating significant differences between clusters. These five clusters are described by 12 pocket and 12 ligand descriptor average values on five star-plots, see Figure 3.C, allowing a useful comparison between the five clusters and the main trends are summarized in Figure 3.D.

Small pocket-ligand pair clusters **a** and **b** are close in terms of pocket and ligand volume but differ in terms of pocket and ligand polarity and pocket narrowness, as detailed in Figure 1. More precisely, clusters **a** and **b** share equivalent smallest volumes for both pockets and ligands (respectively 696 Å and 646 Å for pockets; 190 Å and 196 Å for ligands) and equivalent pocket moments of inertia, pocket hbd%, roughness, planarity and ligand rbond%. However clusters **a** and **b** differ in terms of pocket polarity and ligand polarity (respectively 0.42 *versus* 0.33, for pockets **a** and **b** and 0.45 *versus* 0.25, for ligands **a** and **b**), pocket hba%, narrowness and ligand PSA, LogP and hbd%, see Figure 1. This highlights that different polarity values of small pockets lead into different ligand profiles: more polar pockets with high HBA% tend to bind highly polar ligands associated to weak LogP values, high PSA, HBA% and HBD% values (cluster **a**) while pockets with weak narrowness values tend to bind less polar ligands (cluster **b**).

Large pocket-ligand pair clusters **d** and **e** exhibit also some correspondences between geometrical and physico-chemical pocket and ligand properties. It appears that different shapes of large pockets result in different ligand polarity and geometry. Large pockets share equivalent volume, polaritie, hbd% and hba% values but can differ in shape: moments of inertia, planarity, narrowness and roughness. Interestingly, different shapes of large pockets correspond to different ligand properties such as polarity PSA, LogP but also in terms of moments of inertia and rbond%. Large pockets of cluster **e** are planar, narrow and rough and tend to bind small polar ligands with low rbond% values. So in addition to the observation of Pettit and Bowie [25] mentioning that roughness is necessary for binding in small functional sites (because the required contacts need to be squeezed into a small volume), our classification suggests that large, planar and narrow pockets also need some roughness to be able to bind less polar ligands.

**Figure 4:**
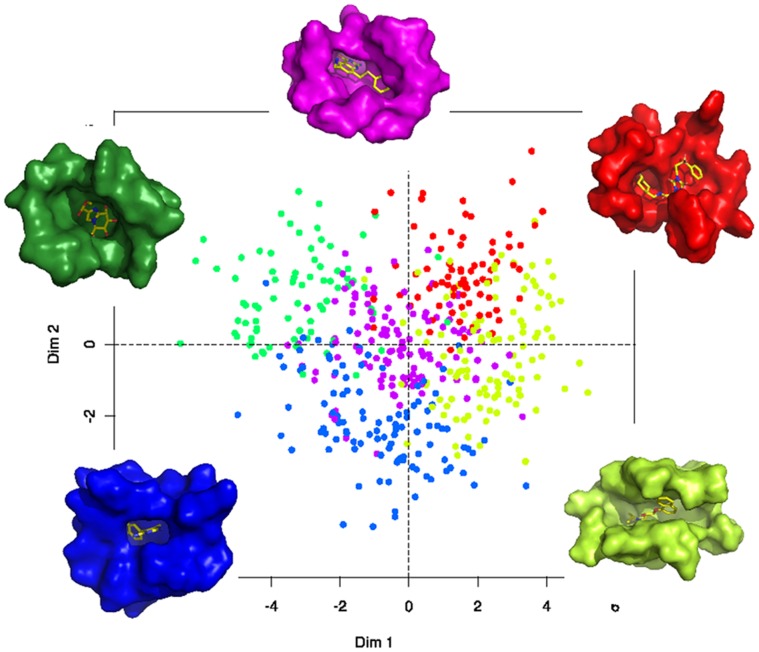
PCA and representatives of five pocket-ligand pair illustrations. Five pocket-ligand pair clusters are illustrated by showing the pair closest from the pocket and ligand average values of each pair. Each pair is coloured according to the clustering of Figure 3.

Cluster **c** is distinguishable from the four other clusters as it presents significant descriptors on average with the other clusters but it has no particular trend for both pocket and ligand properties: all descriptor values are in the middle of the scale, that is closed from the overall values, see Figure 1. In the first PCA plane (see Figure 4.A) cluster **c** is in the center of the plot corresponding to medium values of descriptors, see Figure 1.

Then, in the next step of clustering, this cluster **c** is split into two sub-clusters of similar size with one corresponding to more planar and narrow pockets with low hba% binding larger ligands with higher rbond%. The split of cluster **e** results in two subsets of large pockets and ligands, which the smaller one corresponds to larger pockets binding larger and more polar ligands. Finally, the cluster **b** is splitted in two clusters, which one groups few polar pockets and ligands.

In this work, we want to define different clusters of protein-ligand pairs, which would be useful for a better understanding of protein and ligand matching. There were two constraints: (i) define clusters with specific properties providing interesting knowledge going beyond obviousness and (ii) obtain representative clusters with sufficient members allowing the extraction of information profile. Hence, we considered five clusters, as illustrated on Figure 3.B to catch some general tendencies and to keep sufficient pairs in each cluster. Indeed, the tree cutting leading to five clusters corresponds to a visually clear distance between clusters and the obtained clusters contain from 75 to 118 complexes (see Figure 1), which we consider to be reasonable in terms of occurrence. Figure 4 presents the projection of the five clusters on the first PCA plane and a representation of the average complex of each cluster. We observe that the five clusters are well illustrated on the PCA plane.

From now on and for the subsequent analyses, we consider those five clusters as our reference to test if it is possible to extract useful knowledge for pocket-ligand pair study.

### Characteristics of Pocket-ligand Pair Clusters

First, to check the robustness of these five clusters, it has been compared to the robustness of clusters obtained by random permutations. To do that, we performed a comparison between the robustness of the repartition of pocket-ligand pairs in the five clusters of the classification obtained using 90% of the data *versus* the stability of the five clusters obtained by random permutations using 90% of the data, see section Material & Methods. We obtain an average stability of 74.4% for the five clusters *versus* an average stability of 30.5% for the five clusters obtained by random permutation. Hence, this result highlights the robustness of the classification, which is significantly higher than what would be expected on not meaningful classifications.

**Figure 5:**
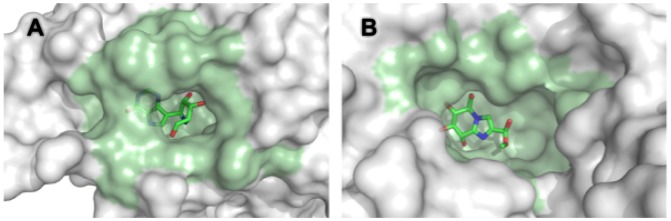
Pairwise percentage of sequence identity computed between all protein chains and Tanimoto coefficient similarity between all ligands. Proteins and ligands are ordered according to order of the five pocket-ligand clusters. The average protein pairwise percentage of sequence identity (values horizontally written) and the average Tanimoto coefficient similarity are also indicated (values vertically written) for the five pocket-ligand pair clusters.

Secondly, we assessed the diversity of proteins and ligands within clusters, in order to ensure that clusters represent a meaningful classification of the pocket-ligand space without an over-representation of some complexes, proteins or ligands. To investigate the relationship between pocket-ligand pair, protein and ligand similarity, we generated a matrix that crosses the similarity of proteins, quantified by the protein pairwise percentage of sequence identity with similarity of associated ligands, quantified by Tanimoto coefficient, and ordered related to the pocket-ligand pair clusters, see Figure 5. Protein redundancy and ligand similarity are low, as shown by the similarity indices distribution provided in Figure S2.I, II. This low protein redundancy is confirmed within each cluster, as illustrated in Figure 5, resulting in an average of 21% of proteins pairwise identity percentage for each cluster. Similarly, the ligand similarity is low in each cluster with an average Tanimoto coefficient of 0.4 between ligand pairs. These results confirm that different pocket-ligand pair clusters are not globally explained by high ligand or protein redundancy and that pocket-ligand pair proximity can be provided from recognition pocket and ligand characteristics.

**Figure 6:**
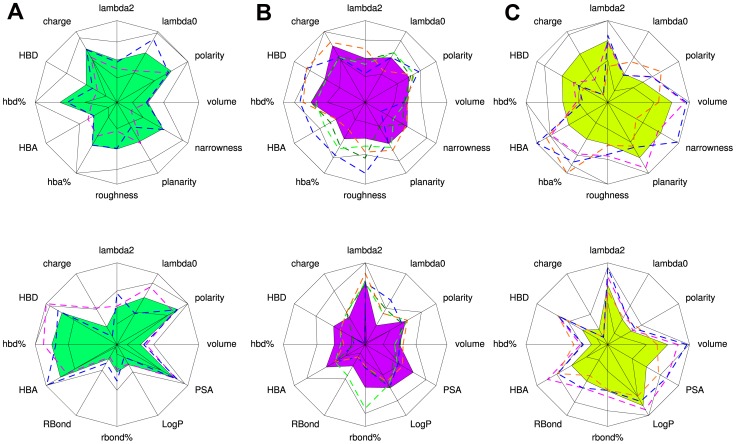
Representation of pocket and ligand properties for 9 pocket-ligand pairs seen in 3 different clusters. (A) Average pocket and ligand profiles of cluster a are represented on star plot. Dashed lines represent pocket and ligand properties of complexes beta-glucosidase bound to IMH ligand (pdb code 1B8O, magenta dashed line) and purine nucleoside phosphorylase bound to GI1 ligand (pdb code 2J7D, blue dashed line). (B) Average pocket and ligand profiles of cluster c are represented on star plot. Dashed lines represent the pocket and ligand properties of 4 cAMP-dependent protein kinases bound to M77 ligand (pdb code 1Q8W, blue dashed line), to 1QP ligand (pdb code 1YDR, dark green dashed line), to 1QS ligand (pdb code 1YDS, green dashed line) and to HFS ligand (pdb code 2ERZ, orange dashed line). (C) Average pocket and ligand profiles of cluster e are represented on star plot. Dashed lines represent the pocket and ligand properties of 3 cAMP-dependent protein kinases bound to BD2 ligand (pdb code 1RE8, magenta dashed line), to B1L ligand (pdb code 1REJ, orange dashed line) and to R69 ligand (pdb code 1XH9, blue dashed line).

Interestingly, this classification is able to group similar pocket-ligand pairs corresponding to different proteins and ligands. For instance, beta-glucosidase A (pdb code 1B8O) and purine nucleoside phosphorylase (pdb code 2J7D) present only 15.3% of sequence identity and bind relatively different ligands (Tanimoto coefficient of 0.22). However, pockets of these two different proteins belong to cluster **a**, corresponding to small highly polar pockets and ligands. Figure 6.A represents the average pocket and ligand profiles of cluster **a** and the pocket and ligand properties of proteins 1B8O and 2J7D. We observe that the pockets of 1B8O and 2J7D are small (a volume of 681 Å and 668 Å, respectively), polar (a polarity of 0.40 for both) and bind small and polar ligands (ligand volume of 216 Å and 205 Å, and polarity ligand of 0.42 and 0.44, respectively) that corresponds to the highlighted properties of pockets and ligands of cluster **a**. This example highlights than two different proteins can have pocket-ligand pairs with several close pocket and ligand properties, resulting in their belonging to the same cluster.

**Figure 7:**
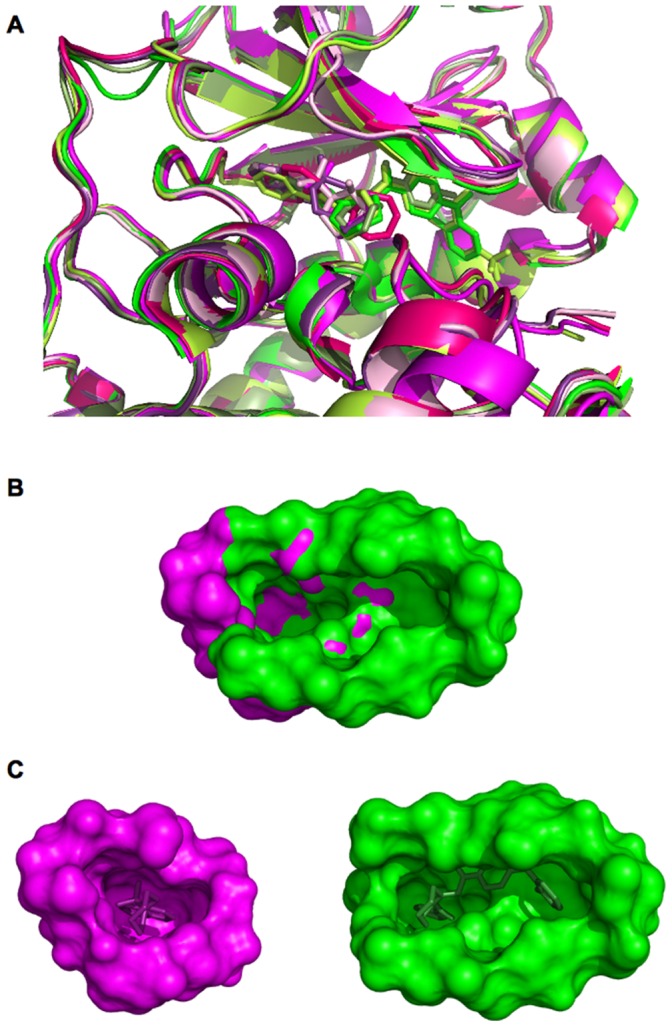
Pocket-ligand pairs extracted from 7 cAMP-dependent protein kinase complexes assigned into two clusters c and e. (A) Superimposition of the 7 cAMP-dependent protein kinase structures using PyMol software. 4 kinases (pdb code: 1Q8W, 1YDR, 1YDS, 2ERZ), with pocket-ligand pairs assigned to cluster c are represented in different magenta. 3 kinases (pdb code: 1RE8, 1REJ, 1XH9) with pocket-ligand pairs assigned to cluster e are represented in different green. Proteins and ligands are represented in cartoon and stick mode, respectively. (B) Superimposition of pockets extracted from the pocket-ligand pairs of pdb files 1Q8W (in magenta) and 1RE8 (in green) are coloured according the cluster that they belong. Pockets are represented in surface. (C) Illustration of the superimposed pocket-ligand pairs extracted from the pdb files 1Q8W (in magenta) and 1RE8 (in green), coloured according the cluster that they belong. Pockets and ligands are represented in surface and stick mode, respectively. Pictures are obtained by PyMol software.

Moreover our classification is able to take into account the fact that the profile of a pocket can change according to the ligand type. This is illustrated using the 7 cAMP-dependent protein kinases present in our dataset: 4 bovin kinases (pdb codes: 1Q8W, 1XH9, 1YDR and 1YDS, UniProt Id: P00517) and 3 mouse kinases (pdb codes: 1RE8, 1REJ and 2ERZ, UniProt Id: P05132). Figure 7.A presents a superimposition of these 7 proteins (a global RMSd of 0.41 Å with more than 98% of sequence identity between the 7 kinases). In our classification these 7 pocket-ligand pairs are assigned into two clusters **c** and **e**. This is explained by the fact that ligands of cluster **e** are larger than ones of cluster **c**, affecting thus the pocket estimation and properties, see Figure 7. Figure 6.B, C illustrate the average pocket and ligand profiles of two clusters **c** and **e** and the pocket and ligand properties associated to the 7 kinases. These kinase pockets are close in terms of polarity values (around 0.38) but differ in terms of volume values (1347.0 Å for cluster **e** *versus* 863.7 Å for cluster **c**). They bind different ligands in terms of volume and polarity: larger and more polar for cluster **e** than for cluster **c** (volume of 374.7 Å *versus* 242.0 Å and polarity of 0.24 *versus* 0.29, respectively). These results highlight that the different ligand profiles of clusters **c** and **e** correspond to different pocket profiles of protein kinases, suggesting different binding modes of protein kinases according the ligand types.

Overall, this analysis highlights strong correspondences between the two interacting partners. Moreover, taking into account a large number of descriptors allows the identification of five pocket-ligand pairs clusters, for which a particular pocket and ligand descriptor profiles can be defined. For instance, large and polar pockets with different shape bind different ligand profile. Thus, in addition to classical trends concerning pocket and ligand volume and polarity, this investigation reveals that combining more information such as pocket shape or roughness can help to determine a preferential profile for ligands. Finally, this classification procedure allows the characterization of 5 clusters grouping pocket-ligand pairs with similar physico-chemical and geometrical profiles. Examples illustrate that pocket-ligand pair clusters can group different protein families and one family can be distributed on different clusters, suggesting that this join complex classification enlarges the notion of protein family.

### Prediction of Pocket-ligand Pair Clusters

We have shown that our classification allows a definition of different pocket-ligand pair profiles. Next, we focus on the possibility of predicting some pocket-ligand pair characteristics for a new partner (either complex or pocket or ligand) through its cluster prediction. Our approach is based on the idea that if a pocket-ligand pair is assigned to one cluster, information about the potential partner profile can be derived from the average characteristics of the pockets and ligands belonging to the assigned cluster.

As the hierarchical classification does not allow assigning a new individual to the obtained clusters, a statistical model has been developed by using proximities between learning pocket-ligand pairs through *k*-nearest neighbor. In order to evaluate the global feasibility of this method, we tested it on both pocket and ligand pair information before more interestingly, applying it to pocket only or ligand only information.

#### Cluster prediction using *pocket-ligand* pair model

In a first step, we developed a method to predict the pocket-ligand pair cluster for a given pocket-ligand pair. Our model is based on (i) the projection of the pocket-ligand pair to be predicted, named *new pair*, in the “*optimal pocket-ligand space*”, (i.e. 14 principal components computed using both pocket and ligand descriptors), and on (ii) a *k*-nearest neighbors method that determines the majority cluster assignment within the closest pocket-ligand pairs of the *new pair*. Here, neighbor pocket-ligand pairs refer to the pocket-ligand pairs with the smallest Euclidean distances with the *new pair* (computed on the *optimal pocket-ligand space*).

We firstly had to determine the number of neighbors *k* used in the k-nearest neighbors by running 100 simulations of a 10-fold cross-validation (see Methods). Table 1 presents the repartition of the obtained optimal number of neighbor *k* during these 100 simulations. For *pocket-ligand pair* model, we chose a number of neighbors of *k* = 3 as it is the optimal in half of the cases. The resulting *k*-nearest neighbors model are summarized in Table 2. We observe that the well-predicted rates are excellent: 90.3% of good prediction on average. In fact, the values range from 88.2% for the less specific cluster **c** to 91.1% for cluster **e**. Using a double cross-validation (see Methods), 78% of simulations have an optimal *k* of 3, (results not shown), and we obtained a global well-predicted rates of 89.6%, range from 86.3% for cluster **c** to 91.6% for cluster **e**, close to results obtained using a simple cross-validation, see Table 2.

**Table 1:** Number of simulations with optimal number of neighbor *k* for the three models *(pocket-ligand pair, pocket-only, ligand-only).*

Number of neighbors	2	3	4	5	6	7	8	9	10	11	12	13	14	15	16	17	18	19	20
*Pocket-ligand pair*		**50**	12	5	1	15	8	9											
*Pocket-only*	5	**82**	9	4															
*Ligand-only*		1	6	3	6	**19**	13	7	5	8	7	6	6		4	5	2	1	1

This was performed on k-nearest neighbor prediction approach using 100 simulations of a 10-fold simple cross-validation, testing *k* values from 2 to 20. The optimal is (*k* = 3) for *pocket-ligand pair* models on 50/100 simulations, (*k* = 3) or *pocket-only* models on 82/100 simulations, (*k* = 7) for *ligand-only* models on 19/100 simulations.

**Table 2:** Pocket-ligand pair cluster prediction results (in percentage) using simple and double cross-validations.

			Clusters				
Models	Cross-validationtype	Overall	a	b	c	d	e
*Pocket-ligand pair*	Simple *(k = 3)*	90.3	82.2	85.3	88.2	91.1	91.0
	Double	89.6	91.2	88.6	86.3	91.1	91.6
*Pocket-only*	Simple *(k = 3)*	63.7	52.6	60.5	58.4	69.0	68.6
	Double	60.3	37.7	62.2	58.7	73.7	65.2
*Ligand-only*	Simple *(k = 7)*	74.8	80.6	66.1	63.7	82.3	76.2
	Double	73.6	90.8	68.2	63.5	84.0	71.6

Ten-fold simple and double cross-validation results obtained on 100 simulations based on *pocket-ligand pair* models, *pocket-only* models and *ligand-*only models using k-nearest neighbors method with *k = 3, k = 3* and *k = 7* neighbors.

These rates are calculated over all pairs with no missing values, that is over 469 pairs instead of 483.

Hence, the high well-predicted rates for *pocket-ligand pair* model suggest that our method could provide relevant results when predicting the cluster of a new pocket-ligand pair. But it is more interesting to provide knowledge about profile of potential interacting partner when only ligand or pocket is known. This is what is proposed in the following section.

#### Cluster prediction using *pocket-only* and *ligand-only* model

In a second step, we extrapolated the cluster prediction when only one partner (pocket or ligand) is known. In the *pocket-only* model, only pocket descriptors are known and used to predict pocket-ligand pair cluster. “Preferred” ligand profile is then deduced from the predicted cluster. In this case, the global average properties of the unknown partner are computed using the learning dataset to project the known partner in the *optimal pocket-ligand space* (see Methods). First, the optimal number of neighbors *k* is 3, as determined using 100 simulations of a 10-fold cross-validation, see Table 1. The results of the overall well-predicted rates using the *pocket-only* model are presented in Table 2. They are quite good: 63.7% on average with a range of 52.6% for cluster **a** to 69.0% for cluster **d**. The optimal *k* and the rates are confirmed using a double cross-validation, see Table 1.

In the case of *ligand-only* model, the choice of the optimal *k* is less clear, see Table 1. Indeed, the most frequent value (*k* = 7) represents only 20% of the 100 simulations but this optimal is confirmed using a double cross-validation. So the *ligand-only* model is performed using *k*-nearest neighbors with *k* = 7. It results in an average well-predicted rate of 74.8% with a minimum of 63.7% for cluster **c** and a maximum of 82.3% for cluster **d**. The results obtained using a double cross-validation are similar, see Table 1.

In *pocket-only* or *ligand-only* models, the data are artificially projected onto the original *optimal pocket-ligand space* by adding virtual missing partner properties. But, as those properties are the same whatever the new ligand or pocket, the real dimension of the space is strongly reduced. Hence, it is not possible to assume that the cluster separateness observed on the *optimal pocket-ligand space* will be conserved by reducing the space dimension only to pocket or ligand space. However, the satisfying results obtained for both *pocket-only* and *ligand-only* models confirm that despite this information loss, clusters are obviously a little less but still separable.

In the case of *pocket-only* and *ligand-only* models, only half of the information (pocket or ligand descriptors, respectively) is used for the prediction whereas the classification is obtained on pocket-ligand pair descriptors. In 75% of cases, a ligand is classified in the good cluster grouping ligands with similar profile binding pockets with similar profile. Conversely, in 64% of case a pocket is classified in the good cluster grouping pockets with similar profile binding ligands with similar profile. In all cases, we observe clusters that are more ligand-specific than pocket-specific, as the well-predicted rates of prediction is overall (and for each cluster) better for *ligand-only* model than for *pocket-only* model. This can be in agreement with the large plasticity of the pockets and their ability to adapt to the ligand [34].

This method gives valuable information about the second partner of an interaction by providing general profile properties of potential ligands (if the pocket is known for example) and in addition examples of ligands having such properties. Our results show that this approach is promising to propose some pocket or ligand profiling. Given a binding-pocket of a therapeutic target, for which no information is known about ligand capable of binding this target, our tool could help finding some critical physico-chemical parameters of likely binders. However, additional investigations are needed to suggest more detailed profiling of likely ligands at this stage but our preliminary results suggest that our models could be valuable in the early steps of drug discovery or of chemical biology.

## Materials and Methods

We carried out a multivariate analysis over the pocket-ligand pair space as one would anticipate that considering descriptors from both ligand and receptor binding pockets would allow better navigation in that space and extrapolations in either, the pocket space or the ligand space. The first step along this line was to build dataset to determine the most important pocket and ligand descriptors within the pocket-ligand pair space. We then combine some pocket and ligand descriptors to define and characterize pocket-ligand pairs.

### Dataset

To create a large *training* set of pocket-ligand pairs, we initially gathered the *refined* set from the PDBbind database [35], [36] and the Astex test set [37] and selected complexes that contained drug-like ligands (*i.e.* small chemical compounds).

Two protein-ligand complex datasets were compiled for the *training* set. The first one is composed of 560 non-redundant protein-ligand structures, with a resolution better than 2.5 Å retrieved from the *refined* set of the PDBbind database. From these structures, we removed those containing metal ions or cofactors next to the co-crystallized ligand resulting in a selection of 432 structures. The second one is composed of 85 manually curated protein-ligand complexes from the Astex test set. As for the previous set, we removed the structures with some ions or cofactors next to the ligand, resulting in a selection of 51 structures. The resulting *training* set corresponds at the end to 483 protein-ligand structures. All the protein files were prepared by adding hydrogen atoms using the software pdb2pqr [38], [39]. For that study, we decided to remove water molecules from the files.

A description of this dataset is available at the following URL: http://dx.doi.org/10.6084/m9.figshare.662781. This description summarizes, in a table, information about each pocket-ligand pair with from the protein side (i) pdb codes, protein chains, protein sequences and their UniProt Id and for the ligands (ii) smile codes, ligand pdb Id (the name of the HET-group ligand) and their cluster assignation.

The redundancy of the 483 proteins was tested by computing pairwise identity percentages between the crystallized sequences of all protein chains. For the computation of the identity sequence percentage, when a protein has several chains, we conserved only the chain presenting the lower sequence identity percentage with other protein chains. In average the identity percentage between protein chain pairs on the *training* set is only of 17%. As indicated by the percentage identity histogram (see Figure S2.I), only a small number of protein chains presents high similarity, for instance only 1.3% of protein chain pairs exhibit more than 90% of identity.

The chemical diversity of ligands was assessed using a fingerprint-based clustering approach and the Tanimoto coefficient with the MDL Public Keys (MACCS). Starting with the 483 ligands and using a dissimilarity criterion of 0.1 (Tanimoto coefficient), 411 cluster centers were obtained therefore proving the overall diversity of ligands. Ligand similarity was illustrated using Tanimoto coefficient histogram, see Figure S2.II. On average the Tanimoto coefficient between ligand pairs of the *training* set is of only 0.4, only a small part of ligands (0,3%) exhibits high similarity (a Tanimoto coefficient higher than 0.8).

### Definition and Description of the Pocket-ligand Pairs

We thus gathered experimental protein structures co-crystallized with a ligand and developed tools to compute established molecular descriptors on both ligands and protein binding pockets.

#### Pocket definition

Among the numerous methods that exist to detect a binding pocket [40] we decided to use the Protomol utility implemented in the software Surflex [41]–[43] (Version 2.1) because it provides an energy-based definition of a binding pocket. The output file is then used to compute several relevant pocket descriptors. The algorithm of Surflex probes the protein binding-sites using a co-crystallized ligand as reference with three types of molecular fragments (CH4, C = O, N-H). These probes constitute what we will refer here to as “virtual pockets”. “True pockets” are defined by pocket solvent-accessible residues and/or atoms in a radius of 4 Å from any probe. Solvent accessibility was computed via the NACCESS program [44] which calculates the atomic accessible surface defined by rolling a probe of given size around a Van der Waals surface.

#### Pocket descriptors

Based on the current literature, we developed some tools/scripts or used available packages to compute the following standard pocket descriptors on the binding cavities predicted by Surflex: volume (MSMS package implemented in Chimera [45]), surface, compacity (ratio between volume and surface), roughness, shape, planarity, narrowness, moments of inertia, polarity, charge, hydrogen bond acceptors and donors, amino acids and atoms composition. Initially, 24 pocket descriptors were considered.

Polarity was represented by a polarity ratio as in [24]. It ranges from 0 (not polar) to 1 (polar). Roughness [25] represents how rough a pocket is: a high value induces a rough pocket. The pocket shape, Rvs [32] provides an estimation of the surface/volume ratio for a given cavity relative to that of a sphere having the same volume as the cavity. Rvs indicates how spherical a cavity is, *i.e.* the value would be 1.0 for a perfect sphere and would become smaller than 1 as the cavity deviates from a sphere. The three moments of inertia correspond to the eigenvalues of the inertia matrix computed on the virtual pockets. The easiest interpretable values are the smallest and the biggest values among the three. We will refer to these terms as lambda0 (for the smallest value) and lambda2 (for the largest value). The moments of inertia of a virtual pocket with regards to a given axis describe how many probes the pocket has overall and how far each probe is from the axis. Consequently the closest the moments of inertia are one from another, the more spherical the pocket is. And conversely the more lambda0 is different from lambda2, the more cylindrical the pocket tends to be. We also compute the planarity of the binding sites [33]. The planarity ranges from 0 (concave) to 1 (flat). To assess how narrow a pocket is, we computed this value as described in [33]. The narrowness ranges from 0 (full circle) to 1 (line). We also considered the pocket charge computed as the difference between the number of positively charged amino acids and the number of negatively charged amino acids as well as the number and proportion of hydrogen bond acceptors (HBA and hba%) and donors (HBD and hbd%). Finally, the number and proportion of amino acids and atoms were computed.

#### Ligand descriptors

Standard ligand descriptors were chosen for our analysis as they are related to pocket descriptors. The following 20 ligand descriptors were computed: volume, surface, shape, moments of inertia, polarity ratio, molecular weight, polar surface area (PSA), charge, LogP, rotatable bond (RBond and rbond%) (counts and proportion), hydrogen-bond acceptors and hydrogen-bond donors (counts and proportion for both). The ligand descriptors also computed on pockets were computed as described in pocket descriptors section while the remaining ones were computed using the software FAF-Drugs2 [46].

A table of pocket and ligand descriptor values of each pocket-ligand pair of the *training* set and the cluster assignation is available upon request to the corresponding author.

### Multivariate Methods

Multivariate analyses were performed by combining both pocket and ligand descriptors. We first defined the most important pocket and ligand properties of the pocket-ligand pairs and removed redundant information using correlation. We then optimized the pocket-ligand property space and removed redundant information through the use of a Principal Component Analysis (PCA). The second step involved the analysis of the pocket-ligand pairs by using a hierarchical clustering approach.

#### Analysis of pocket-ligand pair descriptors

All redundant pocket descriptors and ligand descriptors were removed. This was performed by grouping descriptors having an absolute value of Pearson correlation coefficient higher than 0.8. We chose one representative by group favoring descriptors computed on both pockets and ligands. For instance, we decided to keep the volume descriptor of the ligand rather than its molecular weight (highly correlated to the volume), because the volume is also computed on pockets. We also computed Pearson correlation coefficients between pocket and ligand descriptors.

To define an *optimal pocket-ligand pair space*, a PCA was used. It projects the standardized data, *i.e.* the pocket-ligand pair descriptors, into a subspace made of orthogonal linear combinations of both the original non-redundant pocket and ligand descriptors, so-called principal components. Data may then be explored in a smaller dimensional space spanning the most informative view according to data variability. To keep maximal information from the descriptors while reducing noise, we then work on 95% of the data variability, which corresponds to the first 14 principal components of PCA space, which defines the *optimal pocket-ligand space*. The principal components can also be interpreted in terms of the original descriptors thanks to correlation studies. This allows us to study the relationships between all descriptors, and in particular between pocket and ligand descriptors. The FactoMineR package [47] as implemented in the software R (R development core team 2008) was used to carry out the PCA.

#### Construction of pocket-ligand pair clusters

This *optimal pocket-ligand space*, i.e. space formed by the 14 first component of the PCA, is then used to cluster pocket-ligand pairs by performing a hierarchical clustering [48]. To perform that, Euclidean distances between all pocket-ligand pairs were computed on the *optimal pocket-ligand space*. Using these distances, a hierarchical classification of pocket-ligand pairs was created that allows us to build embedded partitions by progressively gathering pairs then pair clusters according to Ward metric. This metric is a minimum variance method that aims at finding compact and spherical clusters. It results in a tree representation. This tree helps the visualization of the proximity between pair clusters and allows choosing a number *C* of clusters. The function used to cluster pocket-ligand pairs is the “hclust” function implemented in R.

### Analysis of Pocket-ligand Pair Clusters

#### Characterization of clusters

To characterize the obtained pair clusters and reveal their main properties, we performed an analysis of variance on each descriptor with regards to the *C* pair clusters. This analysis allows the determination whether or not a global difference between the average values of the considered descriptors in the different clusters exists. In such a case, a *post-hoc* Tukey’s Honestly Significant Difference test (Tukey’s HSD test) is applied to find which average values are significantly different from the other ones. The results of these tests allow us to characterize pocket and ligand properties among the *C* clusters.

#### Stability of pocket-ligand pair clusters

The robustness of the clustering (initial classification) was tested by analyzing the stability of the *C* clusters obtained after randomly removing 10% of the data, related to *C* random clusters. The *training* set is randomly partitioned into 10 parts respecting the effectives of the *C* true clusters in the *training set*. PCA based on both pocket and ligand descriptors are performed using the pocket-ligand pairs of 9 parts ( = 90% of the data), named *PCA_90*. Euclidean distances between pocket-ligand pairs on 90% of the data were computed on the 14 principal components of *PCA_90*. Using these distances, the 90% of pocket-ligand pairs were clustered using a hierarchical classification, named *hclust_90*. *hclust_90* is then cut to *obtain C* clusters. The repartition of the *C* clusters in *hclust_90* is then compared to the repartition in *C* clusters obtained on initial classification. To quantify the stability of a cluster *I* from the initial classification, the cluster of *hclust_90* including the biggest number of pocket-ligand pairs from this cluster *I* is identified. The percentage of pocket-ligand pairs found in this cluster is computed related to the 90% of the data considered. For example, 66 pairs of cluster *I* of the initial classification are used to build *hclust_90*. Amongst these 66 pairs, 57 are in the cluster homologous to cluster *I* in *hclust_90*. Thus, we count that 86% (57/66) pairs of cluster *I* are well-classified. We performed that for the *C* clusters and compute the average number of well-classified pairs on the *C* clusters. This complete procedure was run 10 times in order to each part was removed one time of the dataset to construct the *PCA_90* and the hierarchical classification, and this 10-fold procedure was run 100 times. To compare, this complete procedure was run on a random dataset obtained after randomly permutated the cluster assignation of the initial data.

### Cluster Prediction Using Similarity Profile

Once a *C*-cluster classification is established, the next step is to develop a statistical model to predict the cluster of a new pocket-ligand complex or a new ligand or a new pocket. As the hierarchical classification does not allow assigning a new individual to the obtained clusters, we performed cluster prediction using the *k*-nearest neighbor method. First, the cluster prediction was built on the case where we knew both pocket and ligand, named *pocket-ligand pair* model. Then, the performance of this approach is tested in a much more challenging and interesting case: when only one partner (pocket or ligand) of the interaction is known, named *pocket-only* and *ligand-only* models, respectively.

#### Cluster prediction using pocket-ligand pair model

The input of our *pocket-ligand pair* model is the vector of ligand and pocket descriptors for all pocket-ligand pairs. The building of the *pocket-ligand pair* model is based on two steps. Firstly, the pocket-ligand pair to be predicted, named *new pair*, is projected on the *optimal pocket-ligand space* that means the 14 first components of the PCA computed using both pocket and ligand descriptors. Then in a second step, we use a *k*-nearest neighbor approach to predict the cluster of the new pocket-ligand pair. *k*-nearest neighbor approach relies on the pocket-ligand pair neighbors, in the *optimal pocket-ligand space* of the *new pair*, that is to say the *k* closest complexes with regards to the Euclidean distances computed in this space. The cluster assigned to the *new pair* is the one including the majority of neighbors. This *k*-nearest neighbor prediction approach needs the determination of the optimal number of neighbors, named *k*. This was performed by using 100 simulations of a ten-fold cross-validation for testing all *k* values from 2 to 20. In order to increase the representativeness of each fold, for each cross-validation application, the frequency of the *C* clusters into each fold is equivalent to the global frequencies obtained in the initial *C*–cluster classification. The final number of neighbor *k* is the one that maximizes the average well-predicted rate obtained after 100 simulations of a 10-fold of cross-validation. Once this number of considered neighbors is chosen, it is applied on the dataset still using a ten-fold cross-validation run 100 times. Then, to avoid overfitting, our protocol was also performed using a double cross-validation scheme allowing an optimization of parameter *k* and a realistic evaluation of the prediction errors [49].

#### Cluster prediction using pocket-only or ligand-only models

Two other prediction models, which are more interesting for application purposes, named *pocket-only* and *ligand-only* models, were training following the same protocol. The *pocket-only* model could be used to propose a profile of potential ligands for a given a *new pocket*, through the prediction of the pocket cluster. To build this model, we used only the pocket descriptors of the considered dataset and assigned to each pocket a virtual ligand profile computed as the average value of each descriptor obtained on *training* set. This virtual ligand is also assigned to the pocket to be predicted, new pocket. Then, the previous prediction protocol is applied to this pocket-virtual ligand pair dataset. Firstly, the pocket-virtual ligand pair dataset and the new pocket-virtual ligand pair are projected on the *optimal pocket-ligand space*, i.e. on the 14 principal components computed using pocket and ligand descriptors. Then, we applied the k-nearest neighbor approach to predict the cluster of the new pocket. Like in *pocket-ligand pair* model, a 10-fold cross-validation is used and a double 10-folds cross-validation to determine the optimal number of neighbor *k*.

The *ligand-only* model could be used to predict the properties of a potential pocket for a given ligand. In that case, we used the similar procedure than the one used to build the *pocket-only* model by determining virtual pocket profiles, which correspond to the average pocket descriptor values on the *training* set.

### Conclusions

We presented here a new original way to merge binding pockets through pocket-ligand pair space by using multivariate analysis on complementary descriptors of these pocket-ligand spaces. The advantage is to propose a join analysis of these two spaces described by interpretable descriptors, without predefinition of feature vectors representing and simplifying each object, resulting in better understanding about correspondences between pocket and ligand properties. For instance, the polarity of pockets and ligands are clearly correlated for small pockets, while the geometry of large pockets determines their ability to bind ligands with different polarity. The reduced pocket-ligand pair space defined by the PCA constitutes a suitable space to classify pocket-ligand pairs and validates the relevance of our approach to consider pocket and ligand spaces together and not independently.

This analysis provides a useful and detailed classification of a representative sample of pocket-ligand pair space resulting for the dataset considered in five particular types of pocket-ligand pairs, which are statistically different from each other with regard to several pocket and ligand properties. Thus, it suggests that similar ligands, in terms of our descriptors, assigned to the same cluster could be good candidates to interact with similar targets, in terms of our descriptors. An interesting point is that this join pocket-ligand pair classification enlarges the notion of protein family. This is in the line of the increasing observations that a drug can hit more than one target. Thus, this type of classification could help to understand compound binding to off- and anti-targets or for polypharmacology analysis.

Using this classification based on pocket-ligand recovery characteristics, a preliminary prediction model approach was developed to predict some pocket profile when we only have ligand information or conversely, to predict potential ligand profile when we only have pocket information. The prediction models from our classification provide good prediction performance, suggesting that our method constitutes a promising tool for drug discovery or chemical biology although substantial investigations such as to define more precise pocket-ligand profile on larger number of clusters obtained on larger complex dataset, are still required to develop our concept. In the future, we believe that this type of approach could open the way to predict off-target candidates in the case of ligand or pocket not yet complexed.

## Supporting Information

Figure S1 **Correlation graphs between some pocket and ligand descriptors.** (A) Correlation graph between pocket volume and pocket sphericity (p-value = 10^−96^). (B) Correlation graph between pocket volume and pocket roughness (p-value = 10^−10^). (C) Correlation graph between pocket volume and ligand volume (p-value≤10^−100^). (D) Correlation graph between pocket polarity ratio and ligand polarity ratio (p-value≤10^−100^). The colors correspond to the classification, which is described in Figure 3. As indicated by the pairs color, large pockets and ligands (red and yellow pairs) correspond to rather few polar pocket associated to more (red pairs) or less polar (yellow pairs) ligands (Figure D) with weak roughness and sphericity pocket values (Figure B and Figure A). This indicates large pockets and ligands correspond to rather few polar, few roughness and few spheric pocket associated to rather few polar ligands suggesting to perform direct multivariate analysis to propose interpretable analysis combining both pocket and ligand descriptors in a more complex way.
(DOCX)

Figure S2 **Histograms of protein identity or ligand similarity computed on the set of 483 complexes.** (A) The redundancy of the 483 proteins is quantified by the identity sequence percentage between each protein pairwise. (B) Ligand similarity of the 483 ligands is quantified using Tanimoto score histogram.
(DOCX)
