# Supplementary material for: Insights into an Original Pocket-Ligand Pair Classification: A Promising Tool for Ligand Profile Prediction
Source: PLoS One. 2013 Jun 20;8(6):e63730. doi: 10.1371/journal.pone.0063730 (PMC3688729; doi:10.1371/journal.pone.0063730)

Figure S1: **Correlation graphs between some pocket and ligand descriptors.**

(A) Correlation graph between pocket volume and pocket sphericity (p-value = 10^-96^).

(B) Correlation graph between pocket volume and pocket roughness (p-value = 10^-10^).

(C) Correlation graph between pocket volume and ligand volume (p-value ≤ 10^-100^).

(D) Correlation graph between pocket polarity ratio and ligand polarity ratio (p-value ≤ 10^-100^). The colors correspond to the classification, which is described in Figure 2. As indicated by the pairs color, large pockets and ligands (red and yellow pairs) correspond to rather few polar pocket associated to more (red pairs) or less polar (yellow pairs) ligands (Figure D) with weak roughness and sphericity pocket values (Figure B and Figure A). This indicates large pockets and ligands correspond to rather few polar, few roughness and few spheric pocket associated to rather few polar ligands suggesting to perform direct multivariate analysis to propose interpretable analysis combining both pocket and ligand descriptors in a more complex way.


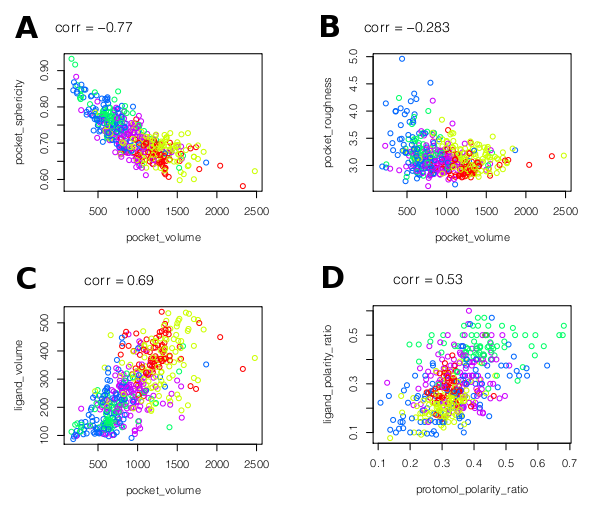

Supplement: Figure S1 — Correlation graphs between some pocket and ligand descriptors. (A) Correlation graph between pocket volume and pocket sphericity (p-value = 10−96). (B) Correlation graph between pocket volume and pocket roughness (p-value = 10−10). (C) Correlation graph between pocket volume and ligand volume (p-value≤10−100). (D) Correlation graph between pocket polarity ratio and ligand polarity ratio (p-value≤10−100). The colors correspond to the classification, which is described in Figure 3. As indicated by the pairs color, large pockets and ligands (red and yellow pairs) correspond to rather few polar pocket associated to more (red pairs) or less polar (yellow pairs) ligands (Figure D) with weak roughness and sphericity pocket values (Figure B and Figure A). This indicates large pockets and ligands correspond to rather few polar, few roughness and few spheric pocket associated to rather few polar ligands suggesting to perform direct multivariate analysis to propose interpretable analysis combining both pocket and ligand descriptors in a more complex way. (DOCX) [file pone.0063730.s001.docx]
