# Supplementary material for: Insights into an Original Pocket-Ligand Pair Classification: A Promising Tool for Ligand Profile Prediction
Source: PLoS One. 2013 Jun 20;8(6):e63730. doi: 10.1371/journal.pone.0063730 (PMC3688729; doi:10.1371/journal.pone.0063730)
Supplement: Figure S2 — Histograms of protein identity or ligand similarity computed on the set of 483 complexes. (A) The redundancy of the 483 proteins is quantified by the identity sequence percentage between each protein pairwise. (B) Ligand similarity of the 483 ligands is quantified using Tanimoto score histogram. (DOCX) [file pone.0063730.s002.docx]

Figure S2: **Histograms of protein identity or ligand similarity computed on the set of 483 complexes.**  (A) The redundancy of the 483 proteins is quantified by the identity sequence percentage between each protein pairwise. (B) Ligand similarity of the 483 ligands is quantified using Tanimoto score histogram.

**A B**
